# Spatial and Sociodemographic Distribution of Predicted 10‐Year Cardiovascular Disease Risk Among Hypertensive Adults in Bangladesh: Evidence From BDHS 2022

**DOI:** 10.1002/puh2.70463

**Published:** 2026-11-07

**Authors:** Chowdhury Mashrur Mahdee, Anika Tabashsum, Mosiur Rahman, Chowdhury Muhtadee Maheer, Fahim Shahriar, Md. Sarwar Zahan, Md. Mamunur Rashid, Md. Yeasir Arafat

**Affiliations:** ^1^ Department of Population Science and Human Resource Development University of Rajshahi Rajshahi Bangladesh; ^2^ Navy Anchorage School and College Dhaka Bangladesh; ^3^ Department of Political Science University of Rajshahi Rajshahi Bangladesh; ^4^ Institute of Educational Development BRAC University Dhaka Bangladesh; ^5^ Department of Population Science Jatiya Kabi Kazi Nazrul Islam University Mymensingh Bangladesh; ^6^ Department of Economics Islamic University Kushtia Bangladesh

**Keywords:** adapted WHO/ISH‐based risk stratification, Bangladesh, cardiovascular disease risk, hypertension, sociodemographic factors, spatial analysis

## Abstract

Cardiovascular disease (CVD) is a major public health problem in Bangladesh, especially among adults with hypertension, yet the sociodemographic patterning, wealth‐related inequities, and spatial distribution of their predicted CVD risk are poorly understood. Using the 2022 Bangladesh Demographic and Health Survey (2544 adults aged ≥18 years; 1412 in the propensity score‐matched sample), we estimated predicted (not overserved) 10‐year CVD risk among adults with hypertension and examined its sociodemographic correlates, wealth‐based inequalities, and geographical clustering. Risk was categorized as low, medium, or high, using a matrix adapted from the World Health Organization/International Society of Hypertension (WHO/ISH) approach, which requires no cholesterol data, and combines hypertension grade, age, sex, recent tobacco use (within 30 min), obesity, and diabetes. Analyses included descriptive and bivariate tests, ordered logistic regression, propensity score matching to assess covariate imbalance across wealth groups, and Global Moran's I and Getis‐Ord Gi* spatial analyses. Overall, 44.7% had low, 18.7% medium, and 36.6% high predicted risk. Older age, female sex, overweight/obesity, recent tobacco use, and higher household wealth were associated with higher predicted risk, although some associations partly reflect the classification rules rather than true differences in risk. In the matched sample, nonpoor participants were slightly more likely than poor participants to have elevated predicted risk (adjusted odds ratio [AOR]: 1.25; 95% confidence interval [CI]: 1.00–1.57), with a stronger association among those with secondary or higher education (AOR: 1.97; 95% CI: 1.09–3.56). Excluding adults under 35 years left most associations and the wealth–education interaction unchanged but altered the education and age effects. Spatial autocorrelation was significant (Global Moran's *I* = 0.133, *p* = 0.027), with hotspots in Comilla, Dhaka, Narayanganj, and Narsingdi. These findings concern predicted risk rather than observed cardiovascular events and may support integrating absolute CVD risk assessment into primary care and geographically targeted prevention for hypertensive adults in Bangladesh.

## Graphical abstract

This study estimated predicted 10‐year CVD risk among hypertensive adults in Bangladesh using BDHS 2022. Sociodemographic inequalities and geographic hotspots highlight priorities for targeted cardiovascular disease prevention.

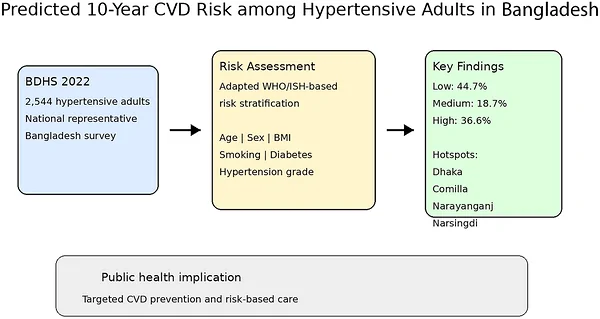

## 1 Introduction

Cardiovascular diseases (CVDs) remain a major public health issue in the world and are one of the largest causes of early mortality and chronic disability globally [1, 2, 3]. Low‐ and middle‐income countries (LMICs) are increasingly bearing a share of the global burden of CVD [1, 4] where health systems often have limited capacity in relation to prevention, early detection, and long‐term management of cardiometabolic risk [4, 5]. The social and economic consequences of CVD in these settings can be substantial and include healthcare expenditure and the loss of productivity and development [5, 6].

Bangladesh is undergoing a rapid demographic and epidemiological transition [3, 7], such as an increase in life expectancy [7, 8], urbanization in large megacities [7, 9], and globalization [9, 10, 11] as well as dietary changes, physical inactivity, and smoking. Along with these population level shifts, there has been a mounting burden of noncommunicable diseases (NCDs) [2, 3, 7] and a rising need for scalable prevention approaches [2, 12]. These socioeconomic factors are seen to be the determinants of cardiovascular risk and outcomes in Bangladesh as well [13, 14, 15] as exposure and resource inequities and access to prevention [12, 13, 14, 15]. It is important to grasp these differences so that fair interventions can be developed that can be beneficial for the people who need it most, whether it is for the person or for the community [12, 14, 15].

The concept of absolute (total) cardiovascular risk [16, 17] continues to be an informative framework for prioritizing preventive interventions in low‐resource settings for the prevention of CVD [16, 18]. Risk prediction tools combine individual risk factors to assign an individual's risk of a cardiovascular event in a given period, usually 10 years [17, 19] and can be used to make more informed decisions than can single risk factor‐based tools [16, 18, 19]. The primary focus of the World Health Organization (WHO)/International Society of Hypertension (ISH) risk prediction charts has been on resource‐limited areas [17, 20], and the aim of developing these charts was to provide a framework for risk‐based prevention and clinical decision‐making [16, 17, 20].

In this regard, attention should be paid to the group of adults with hypertension, because hypertension has been established as one of the most common conditions that contribute to cardiovascular morbidity and mortality [1, 21, 22] and frequently occurs together with other cardiometabolic diseases [22, 23, 24]. Diabetes mellitus is clinically relevant comorbidity [24, 25, 26], and the evidence available to date revealed that the risk associated with hypertension is greatly enhanced by the presence of concurrent diabetes and hypertension [24, 25, 27]. Thus, risk stratification among hypertensive adults can provide information to help target prevention, initiate treatment in a timely manner, and allow for more effective follow‐up [16, 17, 18, 21].

Although the burden of CVD is increasing in Bangladesh, there are important evidence gaps in the distribution of predicted 10‐year CVD risk among adults with hypertension [13, 14] and the extent of predicted 10‐year CVD risk clustering geographically, which could guide place‐based allocation of prevention resources [28, 29, 30, 31, 32, 33, 34, 35, 36, 37, 38, 39, 40].

The findings of this study were derived from nationally representative data collected from the 2022 Bangladesh Demographic and Health Survey (BDHS) [7] to provide evidence for the implementation of risk‐based and equity‐oriented strategy for CVD prevention in Bangladesh, which included (i) estimation of distribution of predicted 10‐year CVD risk among adults with hypertension using an adapted WHO/ISH‐based risk stratification; (ii) examination of sociodemographic correlates of high predicted CVD risk; (iii) comparison of predicted CVD risk between poor and nonpoor respondents, using propensity score matching (PSM) to improve balance across observed covariates; and, finally, (iv) description of the spatial distribution of high predicted CVD risk by measuring geographic clustering and identifying hotspot areas. This is a prediction of the risk category and not actual cardiovascular events.

## 2 Methods

### 2.1 Study Design and Data Source

This analysis was a secondary analysis of data from the 2022 BDHS [7]—a nationally representative cross‐sectional survey to generate population‐level estimates of demographic and health indicators of Bangladesh. The BDHS has standardized questionnaires and biomarker protocols (such as blood pressure [BP] and fasting blood glucose [FBG] measurements) that enable the measurement of cardiometabolic disorders and related risk profiles among adults.

The BDHS 2022 uses a complex sampling design with stratification and multistage cluster sampling, and sampling weights are available for the purpose of drawing unbiased national inferences. In the current analysis, all estimates and inferential models were generated after applying suitable adjustments for sampling weights, clustering, and stratification to account for the BDHS sampling structure [7].

### 2.2 Study Population and Analytic Sample

Adult individuals aged ≥18 years who participated in the BDHS 2022 biomarker measurement component [7, 31] were the target population. The analytic sample was restricted to people who fulfilled the criteria of being hypertensive within the framework of BP classification used in the BDHS (Table 1), as this was the most basic predictor identified in this study. We retained the full ≥18‐year hypertensive age range, rather than restricting it to adults aged 35 years and older, as in a prior application of this approach to BDHS 2011 data in which BP was measured only in adults ≥35 years [41], or to age 40 years and older, as is done in the WHO charts [17, 20], in order to capture the full burden and sociodemographic patterning of predicted risk of CVD in the entire hypertensive population captured by BDHS 2022. This is in line with the study's public health surveillance rather than a clinical risk prediction application. Age is also included in the risk matrix (Table 1) as one of several categorical risk factors—male >55 years, female >65 years—instead of using the continuous variable of age. Because standard 10‐year risk tools are validated primarily in middle‐aged and older adults, predicted risk estimates should be used with caution in younger adults with hypertension (particularly those younger than 35 years of age). To explore this, we performed a sensitivity analysis excluding adults <35 years (Section 2.4.6; Tables S1 and S2).

**TABLE 1:** Study‐defined, adapted World Health Organization/International Society of Hypertension (WHO/ISH)‐based risk stratification matrix used to classify predicted 10‐year cardiovascular disease (CVD) risk among hypertensive adults.

Additional study‐defined risk indicators and diabetes	Grade 1 (SBP 140–159 or DBP 90–99)	Grade 2 (SBP 160–179 or DBP 100–109)	Grade 3 (SBP ≥ 180 or DBP ≥ 110 [mmHg])
No additional indicators	Low risk	Medium risk	High risk
1–2 additional indicators	Medium risk	Medium risk	High risk
≥3 additional indicators, or diabetes	High risk	High risk	High risk

*Note:* This is a study‐defined, adapted risk stratification matrix. It is not a direct application of the validated 2019 WHO South Asia laboratory or non‐laboratory chart [17, 20] and has not been externally validated in Bangladesh or South Asia. It is based on the risk stratification framework of the WHO/ISH hypertension guidelines [41, 42], with the “high” and “very high” categories merged; target‐organ damage and established cardiovascular disease were not available in BDHS 2022. Additional indicators counted were male >55 years, female >65 years, smoking (recent tobacco use), and obesity; diabetes was assigned high risk irrespective of hypertension grade. Original operational labels were low (<15%), medium (15%–20%), and high (>20%); these are category labels rather than calibrated individual event probabilities.

Abbreviations: DBP, diastolic blood pressure; SBP, systolic blood pressure.

The eligibility and measurement of biomarkers were captured in BDHS 2022 for both sexes. Hypertension status was determined using the BP measurement protocol of the BDHS and the standard BP diagnostic criteria. Respondents who met the study criterion for hypertension were retained as the final analytic sample for descriptive, regression, propensity score (PS), and spatial analyses.

### 2.3 Study Variables

#### 2.3.1 Outcome Variable

The study outcome variable was study‐defined and adapted WHO/ISH‐based risk stratification matrix (Table 1) [17, 20, 43] based on predicted 10‐year risk of CVD (including stroke and myocardial infarction), which was classified as low, medium, or high. This is a constructed, predicted risk category derived from survey measurements. It is not an observed cardiovascular event, not a direct application of the validated WHO/ISH risk charts.

BDHS collects information about sex, age, smoking, body mass index (BMI), systolic BP (SBP), and diastolic BP (DBP) and blood glucose level [7, 31]. The systolic and diastolic pressure of the respondents was measured by multiuser upper arm BP monitor UA‐767F/FAC. Three readings of BP were taken at least 5 min apart. BP reading was done and averaged with the second and third readings. Hypertensive participants were defined as those with an average SBP ≥ 140 mmHg or an average DBP ≥ 90 mmHg [7, 21, 22]. A portable HemoCue 201 RT blood glucose meter was used to measure blood glucose level in the BDHS. Diabetes is defined as FBG ≥ 7.0 mmol/L or taking diabetes medication to lower the FBG level [7, 26, 44, 45]. Smoking status was categorized on the basis of the smoking‐related item in the BDHS 2022 biomarker module, which was defined as smoking any tobacco product in the last 30 min. This item reflects the recent tobacco use and is used as a proxy for current smoking. This was the only smoking measure available for all respondents in the biomarker sample, as the BDHS 2022 collected individual‐level interview data only from ever‐married women aged 15–49 years [7] (Woman's Questionnaire) and did not interview men or older women. BMI was used as a measure of obesity, with the Asian cutoff suggested by WHO. A BMI of ≥25 kg/m^2^ was used as a definition for obesity [46].

The classifications of predicted 10‐year CVD risk categories were based on the variables collected in BDHS 2022. The study‐defined matrix (Table 1) is a simple form of the risk stratification framework used by the WHO/ISH hypertension guidelines [42] and is not an application of the WHO/ISH or WHO CVD risk charts [17, 20]. Data from BDHS 2011 has been previously classified into low‐, medium‐, and high‐risk groups using a stratification of adults with hypertension on the basis of a WHO guideline [47]. The primary determinant of risk was hypertension grade (Grades 1, 2, or 3). The presence of other risk factors (age >55 in men, >65 in women, smoking, obesity) moved respondents into a higher risk category, and diabetes assigned respondents to the high‐risk category irrespective of hypertension grade. The “high” and “very high” tiers of the original framework were combined, and target‐organ damage and CVD could not be assessed in BDHS. Cholesterol was not measured in BDHS 2022, so laboratory‐based WHO/ISH charts could not be used; the matrix embraces the logic of non‐laboratory risk assessment that is pertinent in resource‐poor settings [16, 17, 20]. As a study‐defined scheme, the matrix has not been externally validated against observed cardiovascular events in Bangladesh or South Asia. The WHO charts estimate risk from age, sex, SBP, smoking, and laboratory‐based cholesterol and diabetes or BMI (non‐laboratory), whereas the matrix uses categorical inputs. There may, therefore, have been some misclassification of individual risk, and the extent of this misclassification could not be assessed, as there is no cardiovascular outcome data in BDHS. Indirect evidence comes from a prospective cohort of hypertensive adults in rural Ecuador, in which a WHO/ISH stratification using only variables that do not require laboratory tests agreed closely with the laboratory‐based method (weighted *κ* = 0.90) and had similar discrimination (*C* statistic 0.79 vs. 0.74) for cardiovascular events [41].

The CVD risk within the following 10 years was operationalized as low (less than 15%), medium (between 15% and 20%), and high (greater than 20%) [17, 20, 43, 48]; these are category labels and not calibrated individual event probabilities. Risk stratification was done based on the study risk matrix (Table 1), which included hypertension grade and other risk factors, including diabetes status (Table 1) [17, 18, 19, 20].

The associations of age, sex, smoking, obesity, diabetes, and hypertension grade with increased predicted risk are, to some extent, predetermined and are not independent observations, as they are input variables in the risk matrix that defines the outcome. Estimates of these in these regression models are therefore interpreted as being associated with the created risk category rather than as being directly predictive of incident CVD. The characteristics that are not part of the matrix (education, household wealth, place of residence, division, household size, and marital status) are our main inferential interest.

#### 2.3.2 Explanatory Variables

Previous evidence [13, 14, 15, 28, 29] along with availability in BDHS 2022 [7, 31] was taken into consideration while selecting the explanatory variables. These factors encompassed socio‐demographics (age, sex, education level, marital status, household size, wealth quintile [wealth index], and division/region) as well as health‐related characteristics (smoking status, BMI/obesity, and diabetes) [21, 22, 26, 46].

Household socioeconomic position was measured with the DHS wealth index [42], which, typically, is calculated by principal component analysis (PCA) and is usually divided into five quintiles. The definition of wealth, used in the PSM for this analysis, was defined as the analytic specification of this study, where the binary value “poor” was compared with “non‐poor” [32, 33, 34, 35, 42].

### 2.4 Statistical Analysis

#### 2.4.1 Descriptive and Bivariate Analysis

Descriptive data were used to summarize the sample. Questionnaire responses to the ordinal outcome of the risk of CVD (low/moderate/high) were compared using cross‐tabulations against each of the explanatory variables. Pearson's chi‐square tests were used to evaluate the bivariate association between the covariates and the categories of CVD risk; results were considered statistically significant at 5% level.

#### 2.4.2 Regression Modeling

As per WHO/ISH‐based CVD risk studies, age, sex, smoking status, and BMI/obesity were considered covariates along with other sociodemographic and geographic variables. Risk‐algorithm variables (age group, obesity) have also been used as covariates in regression models of predicted CVD risk in previous nationally representative analyses of BDHS data that aimed to characterize which population subgroups carry a disproportionate burden of risk and to inform targeted prevention efforts [47]. The diabetes and hypertension grade, which determine the risk category nearly deterministically, were not added in the regression models (Table 3) and are described in Table 2. Because the risk categories combine these inputs non‐additively (Table 1), the coefficients still show, which subgroups carry a higher predicted risk. However, because these variables also contributed to the construction of the predicted‐risk outcome itself, their associations are interpreted as descriptive correlates of the constructed risk category and not as evidence of independent, causal predictors of incident CVDs (Section 2.3.1). The outcome variable was an ordinal measure, so ordered logistic regression was used to test the relationship between the explanatory variables and the increase in CVD risk levels. Modeling was carried out for unadjusted and multivariable adjusted analyses. Odds ratios (ORs) and 95% confidence intervals (CIs) were calculated for the results of the models, and significance was determined at *p* < 0.05. All regression analyses were weighted using BDHS weights and adjusted for clustering and stratification for national representativeness.

**TABLE 2:** Adapted predicted cardiovascular disease (CVD) risk categories across sociodemographic characteristics before and after propensity score matching: Bangladesh Demographic and Health Survey (BDHS) 2022.

Characteristics	Before matching					After matching
	*n* (%)	Adapted predicted CVD risk category, % (95% CI)				
		Low	Medium	High	*p* value	*n* (%)
**Total**	2544	44.70 (42.36, 47.07)	18.67 (16.96, 20.51)	36.63 (34.37, 38.94)	—	1412
**Age**						
<35	372 (14.62)	66.82 (60.85, 72.29)	12.42 (8.92, 17.05)	20.75 (16.29, 26.06)	<0.001	223 (15.79)
35–54	1046 (41.12)	46.83 (43.44, 50.25)	15.15 (12.92, 17.68)	38.02 (34.65, 41.51)		510 (36.12)
55–69	770 (30.27)	34.50 (30.62, 38.59)	23.85 (20.53, 27.52)	41.65 (37.57, 45.85)		455 (32.22)
70 and above	356 (13.99)	38.14 (32.53, 44.10)	24.16 (19.71, 29.26)	37.69 (32.33, 43.37)		224 (15.86)
**Sex**						
Male	928 (36.48)	45.36 (41.80, 48.96)	21.99 (19.09, 25.20)	32.65 (29.45, 36.02)	0.002	482 (34.14)
Female	1616 (63.52)	44.33 (41.41, 47.28)	16.76 (14.78, 18.93)	38.92 (36.08, 41.83)		930 (65.86)
**Body mass index (BMI)**						
Underweight	223 (8.77)	54.05 (46.54, 61.39)	21.35 (16.08, 27.78)	24.59 (18.78, 31.51)	<0.001	124 (8.78)
Normal	750 (29.48)	50.35 (46.18, 54.51)	19.26 (16.23, 22.70)	30.39 (26.70, 34.35)		501 (35.48)
Overweight	481 (18.91)	48.48 (43.61, 53.37)	15.33 (11.90, 19.52)	36.20 (31.49, 41.18)		263 (18.63)
Obese	1090 (42.85)	36.96 (33.74, 40.30)	19.14 (16.63, 21.92)	43.90 (40.50, 47.37)		524 (37.11)
**Smoked tobacco within 30 min before blood pressure measurement**						
No	2302 (90.49)	47.15 (44.69, 49.62)	16.39 (14.67, 18.27)	36.46 (34.04, 38.94)	<0.001	1274 (90.23)
Yes	242 (9.51)	20.20 (15.26, 26.24)	41.52 (34.73, 48.66)	38.28 (31.66, 45.36)		138 (9.77)
**Diabetes**						
No	1754 (68.95)	63.76 (61.11, 66.33)	26.63 (24.29, 29.10)	9.61 (8.24, 11.19)	<0.001	1038 (73.51)
Yes	790 (31.05)	—	—	100		374 (26.49)
**Hypertension**						
Grade 1	1585 (68.47)	71.25 (68.47, 73.88)	6.14 (4.93, 7.61)	22.61 (20.25, 25.17)	<0.001	894 (67.93)
Grade 2	513 (22.16)	—	73.08 (68.35, 77.35)	26.92 (22.65, 31.65)		299 (22.72)
Grade 3	217 (9.37)	—	—	100		123 (9.35)
**Level of education**						
No education	918 (36.08)	43.92 (40.13, 47.77)	22.67 (19.85, 25.77)	33.41 (29.82, 37.20)	0.006	590 (41.78)
Primary	632 (24.84)	44.55 (40.44, 48.73)	16.66 (13.61, 20.23)	38.79 (34.74, 43.01)		391 (27.69)
Secondary and above	994 (39.07)	45.62 (41.90, 49.39)	15.88 (13.45, 18.65)	38.50 (34.93, 42.20)		431 (30.52)
**Wealth index**						
Poor	1031 (40.53)	50.52 (47.02, 54.01)	21.22 (18.62, 24.08)	28.26 (25.25, 31.49)	<0.001	706 (50.00)
Middle	1069 (42.02)	43.70 (40.13, 47.33)	17.50 (15.01, 20.30)	38.81 (35.42, 42.30)		706 (50.00)
Rich	444 (17.45)	31.65 (26.10, 37.78)	14.91 (11.62, 18.94)	53.44 (47.58, 59.21)		
**Division of residence**						
Barishal	250 (9.83)	44.29 (37.49, 51.31)	21.60 (16.31, 28.04)	34.11 (28.18, 40.58)	0.002	147 (10.41)
Chattogram	357 (14.03)	42.13 (36.57, 47.90)	15.75 (12.37, 19.84)	42.12 (36.35, 48.12)		232 (16.43)
Dhaka	316 (12.42)	39.60 (33.78, 45.72)	15.97 (12.33, 20.42)	44.44 (38.41, 50.63)		136 (9.63)
Khulna	363 (14.27)	44.43 (38.99, 50.01)	22.40 (17.79, 27.80)	33.18 (28.54, 38.17)		218 (15.44)
Mymensingh	243 (9.55)	45.65 (38.47, 53.02)	22.29 (18.12, 27.10)	32.06 (25.04, 39.99)		133 (9.42)
Rajshahi	394 (15.49)	51.19 (44.14, 58.19)	19.73 (14.97, 25.56)	29.07 (23.83, 34.94)		216 (15.30)
Rangpur	350 (13.76)	49.39 (43.70, 55.10)	18.94 (14.49, 24.37)	31.67 (26.60, 37.21)		188 (13.31)
Sylhet	271 (10.65)	43.15 (37.59, 48.89)	18.74 (14.28, 24.21)	38.11 (31.92, 44.71)		142 (10.06)
**Region of residence**						
Urban	987 (38.80)	38.74 (34.56, 43.09)	16.42 (13.72, 19.52)	44.85 (40.32, 49.46)	<0.001	382 (27.05)
Rural	1557 (61.20)	47.19 (44.36, 50.03)	19.61 (17.51, 21.89)	33.21 (30.67, 35.84)		1030 (72.95)
**Number of household members**						
1–4	1263 (49.65)	45.34 (42.08, 48.64)	18.29 (15.90, 20.95)	36.37 (33.23, 39.64)	0.374	673 (47.66)
5–6	854 (33.57)	44.65 (40.80, 48.56)	20.34 (17.46, 23.57)	35.01 (31.55, 38.62)		505 (35.76)
7 and above	427 (16.78)	42.74 (36.79, 48.90)	16.26 (12.48, 20.91)	41.01 (35.35, 46.91)		234 (16.57)
**Marital status**						
Never married/widowed/divorced/separated	603 (23.70)	40.21 (35.83, 44.75)	21.05 (17.65, 24.91)	38.74 (34.19, 43.49)	0.074	370 (26.20)
Married	1941 (76.30)	46.06 (43.35, 48.79)	17.95 (16.01, 20.07)	35.99 (33.48, 38.57)		1042 (73.80)

*Note:* In the matched sample, the middle and rich wealth categories were combined into a single nonpoor category. Chi‐square tests were not performed for the matched sample because propensity score matching is intended to balance covariates between groups by design. After matching, balance was evaluated using standardized mean differences (SMDs) rather than *p* values; all 22 displayed covariate categories had post‐match |SMD| <0.10 (Figure 1; Table S3). Percentages in the risk columns are row percentages with 95 confidence intervals; *p* values are from Pearson chi‐square tests (before matching only). The distributions of diabetes and hypertension grade across risk categories are determined by the construction of the risk matrix (Table 1). The tobacco item records smoking within 30 min before blood‐pressure measurement and is not current smoking status. Hypertension‐grade percentages are calculated among respondents with a recorded grade (*n* = 2315 before matching; *n* = 1316 after matching).

Abbreviation: CI, confidence interval.

#### 2.4.3 PSM Analysis

There are observed differences in education, BMI, and place of residence between households in various wealth groups, and these differences may affect the crude comparisons of the risk predicted between the poor and nonpoor households. PSM was therefore used as a complementary analysis to restrict the confounding bias introduced by observed variables and to compare the predicted risk of poor and nonpoor respondents with similar observed characteristics. We estimated the PS (defined as the conditional probability of being in the exposure group [nonpoor] given observed characteristics) using logistic regression [32, 33]. The PS model was adjusted for age group, sex, education, marital status, BMI category, place of residence (urban/rural), and division of residence (urban/rural) because these were available in the BDHS 2022 and their established associations with both household wealth status and cardiovascular risk factors. As the outcome of interest is a constructed predicted risk group based on cross‐sectional data and not an observed cardiovascular event, in this context PSM should be interpreted not as an indicator of a causal effect of wealth on cardiovascular risk but as a means of making a descriptive comparison between the wealth groups.

We conducted the matching by 1:1 nearest neighbor matching with a caliper of 0.1 [33, 35], consistent with established guidance for constraining poor matches and achieving improved balance [33, 35], which yielded 706 matched pairs. The standardized mean differences (SMDs) (shown in Figure 1 and numerically in Table S3) were used to determine covariate balance before and after matching, with an absolute SMD < 0.10 considered to be indicative of acceptable balance [34, 35]. All analysis was conducted using survey weights from BDHS to ensure the analyses were representative at the national level. Thus, ordered logistic regression models were re‐estimated in the matched sample to evaluate correlates of an increasing predicted CVD risk across the covariate distribution within each wealth group. The matched analysis is understood as an association, rather than a causal effect.

**FIGURE 1:**
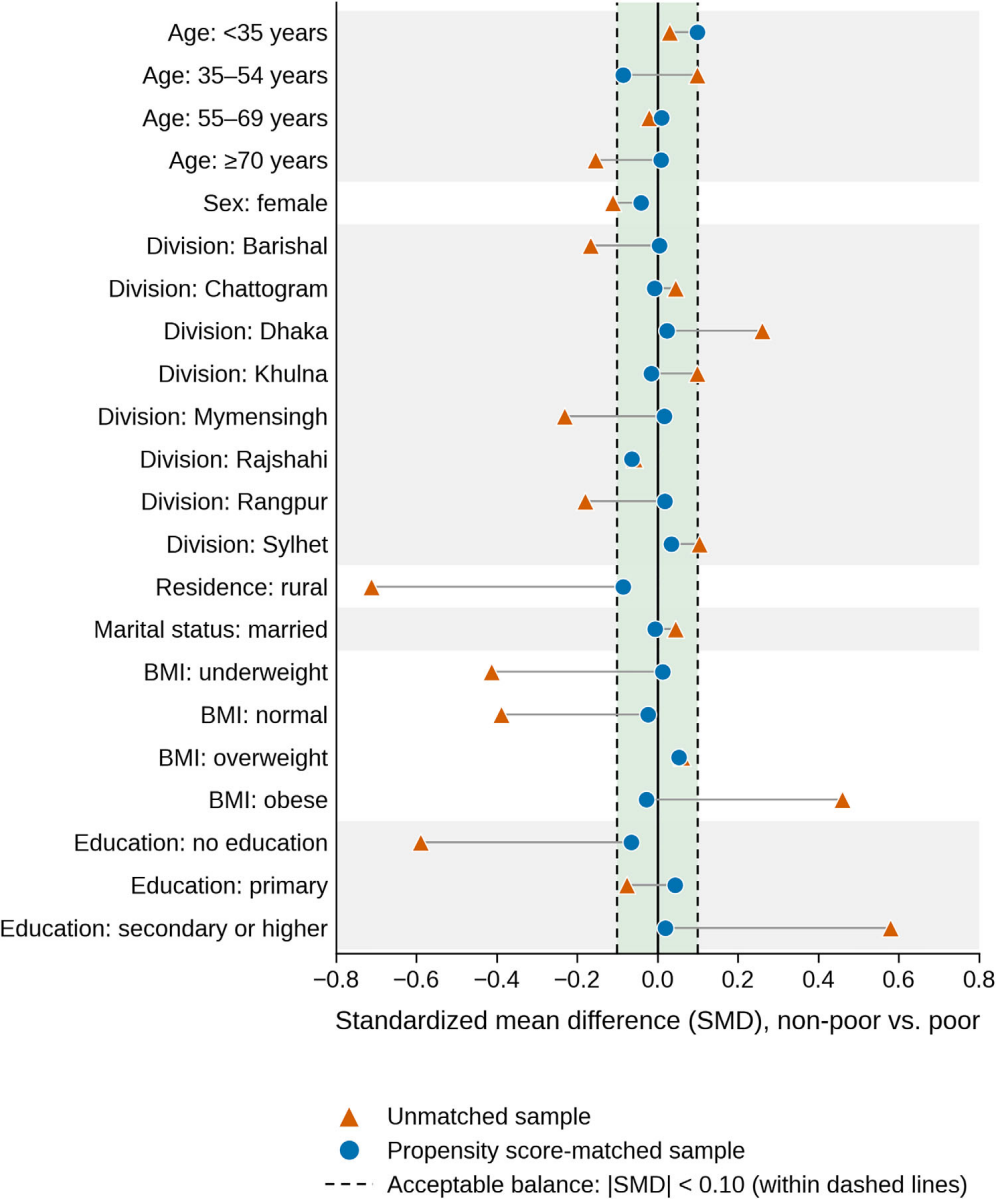
Covariate balance before and after 1:1 nearest neighbor propensity score matching. Triangles show standardized mean differences (SMDs) in the unmatched sample, and circles show SMDs in the propensity score‐matched sample; SMDs compare nonpoor with poor respondents. Dashed vertical lines at −0.10 and +0.10 denote the prespecified acceptable‐balance limits; all 22 displayed covariate categories had post‐match |SMD| <0.10. Numerical SMDs are given in Table S3. BMI, body mass index.

#### 2.4.4 Spatial Analysis

Estimates at the district level were created by combining the results of the BDHS survey clusters within a district boundary [49], and the proportion of adults in the high predicted‐risk category at the district level was examined. To explore the spatial distribution of high predicted CVD risk, we evaluated global spatial autocorrelation using Global Moran's I [36, 37] to test whether district‐level patterns clearly diverged from those expected under a condition of spatial randomness [36, 37]. Local clustering was then measured using the Getis‐Ord Gi* statistics [38, 39], and statistically significant hotspots and cold spots [38, 39] were identified. The district adjacency matrix was created using a neighborhood‐based approach (queen contiguity) [40], and the significance of the hotspot/cold spot was determined by standardized *z*‐scores [38, 39]. The spatial visualization adhered to common mapping standards by showing hotspot, cold spot, and nonsignificant districts using distinct colors [29, 30, 36, 37, 38, 39, 40]. However, BDHS 2022 was not designed to provide estimates at the district level; DHS cluster locations are randomly shifted to preserve confidentiality, and district‐level estimation because of aggregation is prone to ecological bias and the modifiable areal unit problem (MAUP). Spatial results are thus considered exploratory and should therefore be interpreted with caution.

#### 2.4.5 Survey Adjustment and Software

Results were weighted to reflect the complex BDHS survey design that incorporates sampling weights, clustering, and stratification. The statistical analysis was performed by using Stata 16.0 and R.

#### 2.4.6 Sensitivity Analysis

Because standard 10‐year risk tools are validated mainly in middle‐aged and older adults, the descriptive analysis and the ordered logistic regression models were repeated after excluding adults aged <35 years, that is, restricting the analysis to the 35–54, 55–69, and ≥70‐year age groups used in the prior BDHS 2011 analysis [41]. This left 2172 respondents before matching and 1189 in the matched sample. The matched sample was restricted to respondents aged ≥35 years without re‐matching (1412 minus the 223 adults aged <35 years), so the poor and nonpoor groups in Table S1 are no longer equal in size (607 and 582); the results are presented in Tables S1 and S2.

## 3 Results

### 3.1 Descriptive Statistics

Pre‐matching analysis revealed a total of 2544 hypertensive adults (Table 2). Overall, 44.7% were predicted to have low 10‐year CVD risk, 18.7% medium risk, and 36.6% high risk. PSM yielded an analytic sample of 1412 respondents.

Prior to matching, the distribution of predicted CVD risk was very different across several sociodemographic and health‐related factors. Respondents in the <35 age group had the highest percentage in low risk (66.8) and the lowest percentage in high risk (20.8), with the medium‐ and high‐risk profiles significantly higher in the 55–69 and ≥70 age groups, respectively. There were differences between males and females, with more females (38.9%) than males (32.7%) being classified as high predicted risk.

BMI was significantly related to this risk distribution. A higher percentage of obese individuals (43.9) were in the high‐risk group compared to those in the underweight group (24.6). This was also true for recent tobacco use (smoking in the past 30 min); the difference was great: Only 20.2% of those who smoked were at low risk, 41.5% were at medium risk, and 38.3% were at high risk. Diabetes and hypertension grades showed the strongest gradients, as expected because both factors are direct inputs to the risk matrix (Table 1); all respondents with diabetes and all with Grade 3 hypertension belonged to the high‐risk category, whereas 71.3% of those with Grade 1 hypertension were in the low‐risk group (Table 2).

Education, wealth, and division and place of residence were also significantly different. The percentage of people with high predicted CVD risk went up from 28.3 in poor group, 38.8 in middle wealth group, and 53.4 in rich group. The proportion of high risk was higher among urban residents than rural residents (44.9% vs. 33.2%). The division‐wise distribution of the respondents in high‐risk group showed a relatively high percentage in Dhaka and Chattogram divisions and relatively low percentage in Rajshahi and Rangpur divisions. The relationship between household size and marital status with the predicted CVD risk was not significant in bivariate analyses. The poor and nonpoor groups were carefully matched in the matched sample: 706 poor and 706 nonpoor respondents (Table 2). PS matching substantially reduced the SMD for all baseline covariates and resulted in them being close to zero (Figure 1). The absolute SMDs for all 22 covariate categories were <0.10 after matching (maximum 0.099), and 16 of them were <0.05 (Table S3). Prior to matching, the largest imbalances were by place of residence (absolute SMD 0.71), education (0.59 for no education and 0.58 for secondary or higher), and BMI categories (up to 0.46), with smaller imbalances by divisions (up to 0.26). Matching overall reduced the imbalance that was observed and resulted in better comparability between the groups (Figure 1).

### 3.2 Multivariable Analysis Results

Before matching, the predictors associated with increasing predicted 10‐year CVD risk were identified as several factors by ordered logistic regression analysis, and this relationship persisted after the matching (Table 3). One of the strongest correlates remained age. Compared with respondents aged <35 years, those aged 35–54 years (adjusted OR [AOR]: 2.70; 95% CI: 1.99–3.67) and aged 55–69 years (AOR: 4.44; 95% CI: 3.22–6.11) or ⩾70 years (AOR: 4.57; 95% CI: 3.17–6.60) had significantly greater odds of being in a higher CVD risk category in the adjusted pre‐matching model. These associations remained after matching, and the odds were highest in older age groups.

**TABLE 3:** Correlates of a higher adapted predicted cardiovascular disease (CVD) risk category before and after propensity score matching: Bangladesh Demographic and Health Survey (BDHS) 2022.

	Before matching				After matching			
	Adapted predicted CVD risk				Adapted predicted CVD risk			
Characteristics	Unadjusted OR (95% CI)	*p* alue	AOR (95% CI)	*p* value	Unadjusted OR (95% CI)	*p* value	AOR (95% CI)	*p* value
**Age**								
<35	Refs.	—	Refs.	—	Refs.	—	Refs.	—
35–54	2.38 (1.80, 3.17)	<0.001	2.70 (1.99, 3.67)	<0.001	2.29 (1.56, 3.36)	<0.001	2.67 (1.77, 4.01)	<0.001
55–69	3.33 (2.48, 4.47)	<0.001	4.44 (3.22, 6.11)	<0.001	2.85 (1.93, 4.23)	<0.001	3.91 (2.56, 5.97)	<0.001
70 and above	2.86 (2.08, 3.93)	<0.001	4.57 (3.17, 6.60)	<0.001	3.11 (2.09, 4.63)	<0.001	4.79 (3.00, 7.67)	<0.001
**Sex**								
Male	Refs.	—	Refs.	—	Refs.	—	Refs.	—
Female	1.15 (0.98, 1.36)	0.084	1.22 (1.01, 1.46)	0.034	1.34 (1.07, 1.68)	0.011	1.40 (1.09, 1.81)	0.009
**Body mass index (BMI)**								
Underweight	Refs.	—	Refs.	—	Refs.	—	Refs.	—
Normal	1.21 (0.89, 1.65)	0.218	1.23 (0.89, 1.70)	0.214	1.37 (0.92, 2.06)	0.125	1.57 (1.02, 2.39)	0.039
Overweight	1.41 (1.02, 1.96)	0.039	1.55 (1.09, 2.20)	0.015	1.31 (0.83, 2.05)	0.236	1.65 (1.02, 2.66)	0.040
Obese	2.12 (1.58, 2.85)	<0.001	2.31 (1.65, 3.23)	<0.001	1.92 (1.29, 2.86)	0.001	2.83 (1.82, 4.40)	<0.001
**Smoked tobacco within 30 min before blood pressure measurement**								
No	Refs.	—	Refs.	—	Refs.	—	Refs.	—
Yes	1.72 (1.40, 2.12)	<0.001	1.82 (1.45, 2.29)	<0.001	1.98 (1.52, 2.58)	<0.001	2.05 (1.53, 2.76)	<0.001
**Level of education**								
No education	Refs.	—	Refs.	—	Refs.	—	Refs.	—
Primary	1.09 (0.89, 1.34)	0.382	1.22 (0.98, 1.53)	0.078	1.00 (0.78, 1.29)	0.975	1.19 (0.92, 1.56)	0.189
Secondary and above	1.06 (0.87, 1.30)	0.563	1.27 (0.99, 1.64)	0.063	0.78 (0.60, 1.02)	0.070	1.43 (1.04, 1.97)	0.028
**Wealth index**								
Poor	Refs.	—	Refs.	—	Refs.	—	Refs.	—
Middle	1.43 (1.18, 1.72)	<0.001	1.18 (0.97, 1.44)	0.092	1.22 (0.98, 1.53)	0.075	1.25 (1.00, 1.57)	0.048
Rich	2.54 (1.94, 3.32)	<0.001	1.86 (1.37, 2.54)	<0.001				
**Division of residence**								
Barishal	Refs.	—	Refs.	—	Refs.	—	Refs.	—
Chattogram	1.23 (0.88, 1.72)	0.229	1.20 (0.84, 1.71)	0.320	1.12 (0.74, 1.68)	0.601	1.18 (0.76, 1.82)	0.468
Dhaka	1.36 (0.97, 1.92)	0.078	1.18 (0.82, 1.70)	0.363	1.09 (0.67, 1.76)	0.735	1.23 (0.75, 2.02)	0.418
Khulna	0.98 (0.72, 1.33)	0.896	0.92 (0.66, 1.28)	0.602	0.87 (0.58, 1.32)	0.524	0.93 (0.58, 1.49)	0.761
Mymensingh	0.93 (0.64, 1.36)	0.722	1.04 (0.70, 1.55)	0.832	0.99 (0.62, 1.61)	0.998	1.00 (0.60, 1.65)	0.995
Rajshahi	0.78 (0.55, 1.10)	0.156	0.82 (0.56, 1.19)	0.292	0.74 (0.46, 1.17)	0.192	0.81 (0.49, 1.34)	0.416
Rangpur	0.85 (0.62, 1.17)	0.320	0.95 (0.67, 1.35)	0.781	0.96 (0.61, 1.49)	0.841	1.06 (0.65, 1.71)	0.819
Sylhet	1.11 (0.79, 1.55)	0.549	1.12 (0.79, 1.61)	0.518	0.85 (0.55, 1.32)	0.465	0.99 (0.62, 1.59)	0.971
**Region of residence**								
Urban	Refs.	—	Refs.	—	Refs.	—	Refs.	—
Rural	0.66 (0.54, 0.81)	<0.001	0.82 (0.66, 1.02)	0.081	0.77 (0.60, 0.99)	0.046	0.72 (0.55, 0.94)	0.015
**Number of household members**								
1–4	Refs.	—	Refs.	—	Refs.	—	Refs.	—
5–6	0.99 (0.82, 1.19)	0.908	0.91 (0.75, 1.10)	0.323	1.03 (0.81, 1.31)	0.816	0.93 (0.73, 1.20)	0.593
7 and above	1.16 (0.89, 1.52)	0.275	0.95 (0.72, 1.25)	0.707	0.93 (0.66, 1.30)	0.653	0.76 (0.53, 1.08)	0.128
**Marital status**								
Never married/widowed/divorced/separated	Refs.	—	Refs.	—	Refs.	—	Refs.	—
Married	0.83 (0.69, 1.01)	0.068	0.89 (0.70, 1.12)	0.316	0.80 (0.63, 1.02)	0.067	0.87 (0.66, 1.15)	0.338
**Interaction effect**								
Nonpoor (vs. poor) within primary	—	—	—	—	—	—	0.82 (0.50, 1.33)	0.418
Nonpoor (vs. poor) within secondary and above	—	—	—	—	—	—	1.97 (1.09, 3.56)	0.025

*Note:* Middle and rich wealth categories were combined as nonpoor after matching (matched columns: “middle” = nonpoor vs. poor). The tobacco item records smoking within 30 min before blood pressure measurement, not current smoking status. Age, sex, BMI, and this item contributed to outcome construction, so their coefficients are descriptive correlates, not independent predictors of incident CVD. Interaction effects (matched sample only) were adjusted for age, sex, BMI, smoking, division, region, household size, and marital status. PSM and interaction estimates are noncausal and susceptible to residual confounding.

Abbreviations: AOR, adjusted odds ratio; CI, confidence interval; OR, odds ratio; Ref., reference.

Female respondents were significantly more likely than males to be in a higher CVD risk category in both the unmatched analysis (AOR: 1.22; 95% CI: 1.01–1.46) and matched analysis (AOR: 1.40; 95% CI: 1.09–1.81). There was also an obvious dose response with BMI. Before matching, overweight and obese respondents had significantly higher odds of high CVD risk compared to underweight respondents, after controlling for demographics (AOR: 1.55; 95% CI: 1.09–2.20) and (AOR: 2.31; 95% CI: 1.65–3.23), respectively. In the matched sample, the association for obesity was even stronger (AOR: 2.83; 95% CI: 1.82–4.40), and normal BMI (AOR: 1.57; 95% CI: 1.02–2.39) and overweight status (AOR: 1.65; 95% CI: 1.02–2.66) were also associated with significantly higher odds (Table 3).

As predicted, the higher the predicted risk, the more recent the use of tobacco. Respondents who had smoked within the previous 30 min had almost twice the odds of being in a higher CVD risk category in both unmatched (AOR: 1.82; 95% CI: 1.45–2.29) and matched (AOR: 2.05; 95% CI: 1.53–2.76) adjusted models. There was a slightly less robust pattern before matching, but after matching, respondents with secondary education or higher showed significantly higher odds of an elevated predicted risk than those with no education (AOR: 1.43; 95% CI: 1.04–1.97) (Table 3).

### 3.3 Interaction Effect in the Matched Sample

Matched‐sample interaction analysis indicated that the association of wealth status with predicted CVD risk differed by educational attainment (Table 3). The predicted CVD risk was similar for nonpoor and poor respondents with primary education level (AOR: 0.82; 95% CI: 0.50–1.33). However, among those with secondary education or higher, nonpoor respondents had almost twice the odds of being in a higher CVD risk category compared with their poor counterparts (AOR: 1.97; 95% CI: 1.09–3.56). The wealth–risk association thus seemed to be stronger as education increased. This interaction analysis should be viewed as exploratory and should be interpreted with caution.

### 3.4 Spatial Distribution and Clustering of High Predicted CVD Risk in Bangladesh

Figure 2 shows the spatial distribution and clustering of the highest predicted CVD risk in Bangladesh. At 95% confidence level, hotspots of high predicted CVD risk were identified in Comilla, Dhaka, Narayanganj, and Narsingdi, whereas Barishal and Rajbari were found as cold spots at 90% confidence level. The positive spatial autocorrelation (Global Moran's *I* = 0.133, *p* = 0.027) suggests that the distribution of high predicted CVD risk across the districts was nonrandom and geographically clustered in Bangladesh. These findings are exploratory, as district‐level estimates rest on small number of respondents.

**FIGURE 2:**
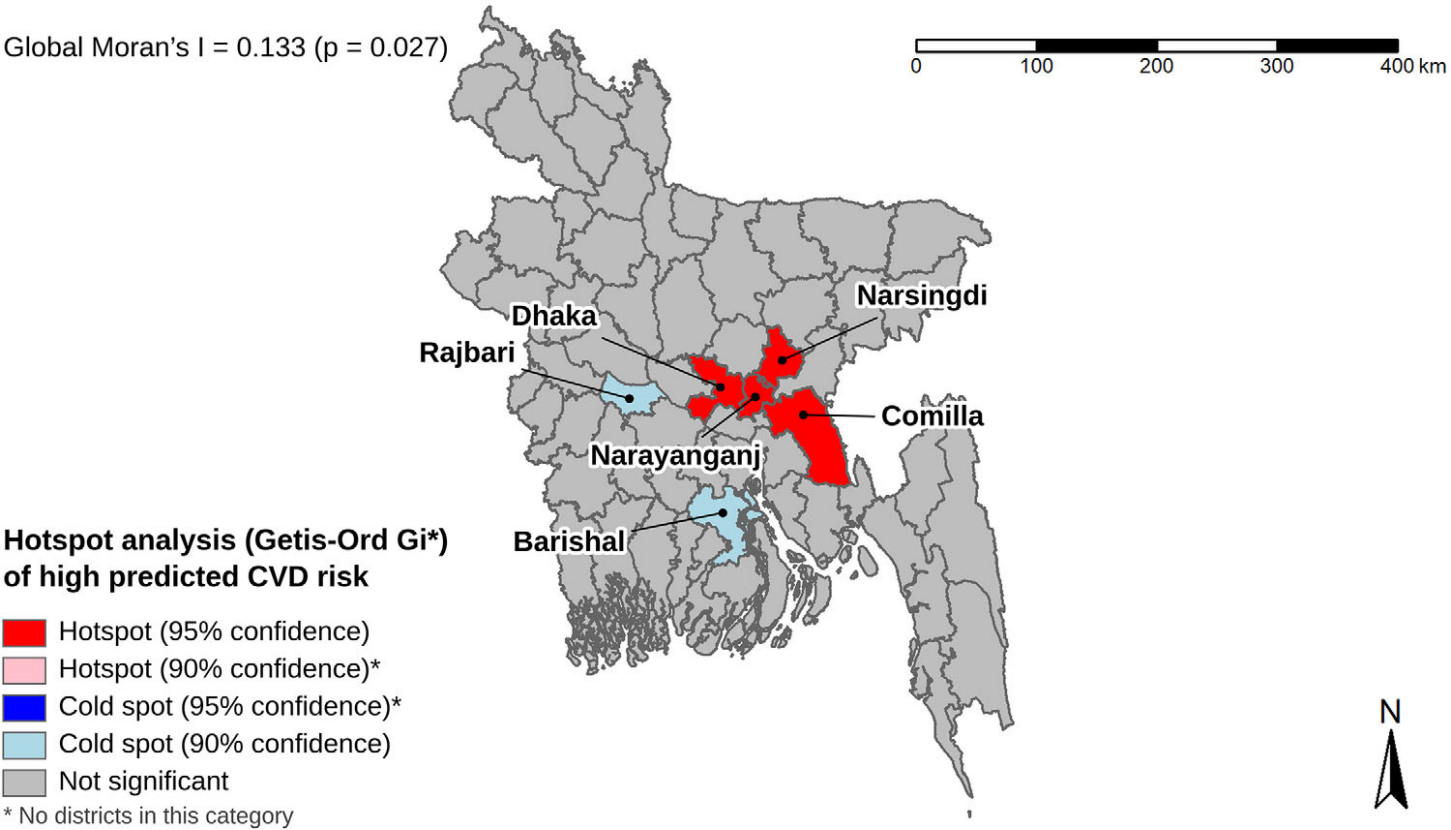
Getis‐Ord Gi* hotspot and cold‐spot classification for the district‐level proportion in the highly adapted CVD risk category. Red and pink denote 95% and 90% hotspots, dark and light blue denote 95% and 90% cold spots, and gray denotes nonsignificant districts; only 95% hotspots (Comilla, Dhaka, Narayanganj, and Narsingdi) and 90% cold spots (Rajbari and Barishal) were observed. Global Moran's *I* = 0.133 (*p* = 0.027). The map does not display district‐specific estimate precision and should be interpreted as exploratory. CVD, cardiovascular disease.

### 3.5 Sensitivity Analysis Excluding Adults Aged <35 Years

After excluding adults aged <35 years, 2172 hypertensive adults remained before matching and 1189 after matching. The distribution of predicted risk showed a shift towards higher categories (41.1%, 19.7%, and 39.2% low, medium, and high risk, respectively; Table S1), compared to that in the full sample (44.7%, 18.7%, and 36.6%). In the ordered logistic regression models (Table S2), older age was also associated with increased predicted risk (adjusted pre‐matching model, reference 35–54 years; 55–69 years, AOR: 1.63, 95% CI: 1.31–2.04; ≥70 years, AOR: 1.65, 95% CI: 1.21–2.25). Obesity (AOR: 2.32; 95% CI: 1.62–3.33 before matching and AOR: 2.75; 95% CI: 1.70–4.46 after matching), recent tobacco use (AOR: 1.72; 95% CI: 1.35–2.20 and AOR: 1.88; 95% CI: 1.38–2.57) and rural residence (AOR: 0.79; 95% CI: 0.63–0.99 and AOR: 0.71; 95% CI: 0.53–0.95) also remained associated with predicted risk, and the wealth–education interaction persisted (secondary or higher education: AOR: 2.38; 95% CI: 1.30–4.36; primary education: AOR: 0.95; 95% CI: 0.57–1.58).

Some estimates were less robust. The female excess was attenuated before matching (AOR: 1.16; 95% CI: 0.95–1.43) but remained in the matched sample (AOR: 1.33; 95% CI: 1.01–1.75). The association between nonpoor status and predicted risk (AOR: 1.06; 95% CI: 0.77–1.47) and the association for secondary or higher education (AOR: 0.97; 95% CI: 0.64–1.47) were no longer statistically significant in the matched sample, although the rich‐versus‐poor contrast before matching remained (AOR: 1.73; 95% CI: 1.23–2.41). The two matched sample estimates were thus influenced by the presence of adults <35 years.

## 4 Discussion

### 4.1 Main Findings

In this nationally representative analysis of hypertensive adults in Bangladesh, more than a third (36.6%) were classified as having high predicted 10‐year CVD risk and about a fifth (18.7%) as having medium predicted risk, indicating a considerable burden of elevated predicted risk in an already vulnerable population. These are the predicted risks as per an adapted WHO/ISH‐based matrix and are not cardiovascular events. Older age and female gender, overweight or obesity, recent tobacco use, higher household wealth, and urban residence were all associated with higher predicted risk of CVD. The associations for age, sex, smoking, obesity, diabetes, and hypertension grade are, by design, part of the developed risk category and should be interpreted as correlates of the created risk category and not as independent risk factors to predict incident CVD. The associations persisted in multivariable models, and, for wealth, following the PSM, indicating that the difference in wealth is related to the observed covariates included in the matching, but that unobserved confounding should not be ruled out and that no causal inference can be made. Furthermore, the interaction analysis showed that the positive relationship between wealth and predicted CVD risk might be stronger among the more highly educated. In a sensitivity analysis excluding adults aged <35 years, the associations for age, obesity, recent tobacco use, urban residence, and the wealth–education interaction were unchanged, but the matched main‐effect estimates for wealth and education were not, so these two estimates should be interpreted with caution. Finally, the spatial analysis revealed that there were geographic hotspots and cold spots within the study district, the hotspots being Comilla, Dhaka, Narayanganj, and Narsingdi, whereas Barishal and Rajbari were the cold spots. All these results indicate social patterning and geographic clustering of expected CVD risk among the hypertensive adults in Bangladesh.

The results provide several possible implications. First, hypertension‐induced cardiovascular risk is not equally distributed across the population of adults but structured by sociodemographic factors, behavioral factors, and sociodemographic factors. Second, the increase in the predicted risk of the nonpoor and urban hypertensive adults is slight and may reflect the epidemiological transition in Bangladesh, where the prevalence of cardiometabolic risk is shifting to sub‐populations that are more vulnerable to exposure to obesogenic environments, lifestyle choices, and shifts in diet. Third, geographic clustering may indicate that there is a need for place‐sensitive and risk‐based preventive interventions at the district level rather than a one‐size–fits‐all national‐level policy; due to the imprecision of district‐level estimates, this should be verified with district‐level inference‐based estimates.

### 4.2 Comparison With Other Studies

These findings align with previous studies in Bangladesh and other LMICs that found cardiovascular risk rises steeply with age [14, 17, 23] and is shaped by coexisting metabolic and behavioral risk factors [17, 23, 24, 25, 26, 27]. Our finding of disproportionately high predicted risk in older hypertensive adults is supported by a national Bangladeshi study that utilized non‐laboratory risk charts from the WHO, showing that risk for CVD was higher with age and differed according to social and behavioral factors [14, 17].

The associations we found between overweight/obesity and smoking are consistent with the literature as a whole [1, 11, 12, 23, 46], but, as stated above, this relationship is partly incorporated in the risk matrix. The trend toward concentrated affluence, among wealthier and urban households, is reflected in overweight/obesity [11, 12, 13, 14, 15] and diabetes [14, 21, 22, 23] and hypertension [28] traits in Bangladesh, which is marking the nutrition and epidemiological transitions [7, 8, 9, 10] in the country. This could account for the marginally higher predicted risk score among nonpoor respondents as compared to poor respondents in our study [12, 13, 14, 15, 28]. This relationship is not in direct contradiction to the classic social gradient of CVD that has been observed in high‐income countries but rather may reflect an intermediate stage in which higher material resources are associated with more calorie‐dense diets, sedentary occupations, and other exposures to cardiometabolic risk factors [8, 9, 10, 11, 12, 15]. Because the data are cross‐sectional and the outcome is an artificial risk category, which means that we cannot make causality conclusions about wealth and risk.

The higher predicted CVD risk among the urban residents compared to rural residents could be a plausible difference in the Bangladeshi context [12, 15, 28]. The prevalence of overweight/obesity, hypertension, and diabetes is higher in urban areas [10, 11, 12, 13, 14, 15, 28], which could be related to variations in diet, occupational physical activity, transport usage, and availability of processed foods [8, 9, 10, 11, 12]. Risk‐factor profiles among hypertensive adults, therefore, could be concentrated in urban environments, but this cannot be claimed given the cross‐sectional design.

The higher predicted risk among women needs to be interpreted with caution and is a matter of equity. In the general adult population, males were found to be at higher risk as per earlier Bangladeshi study based on the non‐laboratory risk charts developed by WHO [14]. This variance may be because we limited our study to hypertensive adults and applied a different risk stratification approach and set of explanatory variables [17, 20]. It can also be due to a less favorable profile of obesity, diabetes, and age‐related risk factors in the case of hypertensive women [12, 24, 25, 26, 27]. The increased risk of higher predicted risk among women in the hypertensive cohort (AOR: 1.22 pre‐ and 1.40 post‐matching) might be explained by the following factors: comorbid risk factors clustering more in women than men with hypertension; differences in healthcare‐seeking or when healthcare seeking occurs; and failure to recognize cardiovascular risk in women in clinical practice. These explanations are tentative and have not been tested with our data. It is important to further explore the pattern and to advocate sex‐sensitive ways of communicating and preventing the risk of developing CVD, including in the target group of women with hypertension. The spatial results are similar to other studies showing geographic variation in cardiometabolic and heart disease burden in Bangladesh [29] and other South and Southeast Asian countries [30]. The district‐level difference reflected a national picture where there was a marked geographical variation of exposure to heart disease with clustering of the exposures in central areas [29], consistent with our hotspots in Dhaka and neighboring districts. This clustering might be because there are common contextual factors such as urbanization, population density, food environments, access to health services, and regional lifestyle [8, 9, 10, 11, 12, 29, 30]. Our findings support the evidence that factors affecting the individual, as well as the geographical location of the high‐risk groups, should be considered in the prevention of CVD in Bangladesh [16, 17, 29]. They should be treated with caution, however, given that these district‐level findings are not planned for in the BDHS 2022 design, small district sample sizes, random displacement of DHS cluster coordinates, and ecological bias and the MAUP associated with aggregation to districts. Hotspots may thus vary under other spatial units and should not be interpreted as individual‐level risk.

Generally, our results are in‐line with evidence that in Bangladesh NCDs are becoming the main contributors to the burden of disease [2, 3, 7, 12]; high BP, high fasting plasma glucose, and smoking are the major drivers of the burden of disease in the country [3, 21, 22, 23, 24, 25, 26]. This clustering of predicted risk in the hypertension adult population is not surprising [17, 20, 21] and underscores the need to go beyond detecting hypertension to a more comprehensive approach of absolute risk assessment [16, 17, 18].

### 4.3 Strengths and Limitations

This study has several strengths. *First*, the data were from the nationally representative BDHS 2022, and the results reflect the population‐level distribution of predicted CVD risk among adults with hypertension in Bangladesh. *Second*, sociodemographic, behavioral, and geographic factors were included in the analysis, and ordered logistic regression was used because the risk outcome was ordinal. *Third*, the risk classification does not depend on cholesterol measurements but on variables routinely collected (BP, age, sex, smoking, BMI, and diabetes status); this pragmatic approach, although a limitation for precision, is a scalable, low‐cost alternative to cholesterol measurement for risk stratification in primary care settings in resource‐constrained countries like Bangladesh and other LMICs [16, 17, 20]. A prospective cohort study of adults with hypertension in Ecuador showed that a WHO/ISH stratification based on key variables was comparable to the laboratory‐based method [51]. *Fourth*, PSM enabled the differences in wealth between poor and nonpoor respondents with comparable observed features to be compared. *Finally*, incorporating spatial autocorrelation and hotspot analysis adds a geographic dimension to the study, by detecting nonrandom clustering and areas where predicted CVD risk is higher.

A few limitations, however, must be noted. *First*, the study was conducted using cross‐sectional survey data, which is not suitable for making causal conclusions and is not suitable for making inferences about the temporality of the associations found; the associations found should be interpreted as correlational rather than causal. *Second*, the outcome is a predicted level of risk instead of an actual cardiovascular event, and there was no data available on cardiovascular events to evaluate the accuracy of the risk levels. The risk matrix is a study‐defined, adapted WHO/ISH‐based scheme that has not been externally validated (although a non‐laboratory WHO/ISH stratification performed similarly to the laboratory‐based scheme in a cohort of hypertensives in Ecuador [41]) and has categorical inputs, so there is likely some misclassification of absolute risk compared with WHO laboratory or non‐laboratory charts; the amount of such misclassification was not quantifiable in these data. *Third*, the outcome was built based on age, sex, smoking, obesity, diabetes, and hypertension grade; associations to these variables are at least partly expected by design (e.g., the very strong associations for diabetes and hypertension Grade 3) and not independent findings. These should be considered markers of the built risk category and not as separate risk factors for incident CVD. *Fourth*, because standard 10‐year CVD risk prediction tools are validated mainly in middle‐aged and older adults, our inclusion of all hypertensive adults aged ≥18 years may affect the calibration and interpretation of predicted risk for younger participants of age, whereas these tools have been validated primarily in individuals aged 40 years and older. A sensitivity analysis excluding adults aged <35 years (Tables S1 and S2) showed that the associations for age, obesity, recent tobacco use, urban residence, and the wealth–education interaction were robust, whereas the matched estimates for household wealth and for secondary or higher education were no longer statistically significant, and the female excess was attenuated before matching; these estimates should therefore be interpreted with caution.

Fifth, although PSM improves balance on observed covariates, it does not remove bias due to unmeasured confounding. The matched estimates may not be interpreted as causal effects of household wealth because residual confounding due to diet, physical activity, use of medication, lipid status, family history of CVD, or access to healthcare may remain. *Sixth*, the measure of smoking available for all respondents in BDHS 2022 was defined as any tobacco use within the past 30 min, because individual interviews were completed only by ever‐married women aged 15–49 years [7]. This item is a proxy for recent use that may not reflect current or long‐term smoking that is relevant for cardiovascular risk; it may have led to under‐identification of smokers and of predicted risk in some respondents. *Seventh*, our spatial analysis was performed at the district level, which is useful for targeting policies but can mask the heterogeneity within districts and can be sensitive to small area instability. BDHS 2022 was not designed to produce district‐level estimates; DHS cluster coordinates are randomly displaced for respondent confidentiality, and district‐level estimates aggregate heterogeneous clusters, so our spatial findings may be subject to ecological bias and the MAUP, and hotspot estimates for districts with small samples should be interpreted cautiously given greater instability. Hotspots should not be interpreted as individual‐level effects. *Finally*, as the study was restricted to adults with hypertension, extrapolation of results to the general adult population in Bangladesh must be done with caution.

## 5 Conclusions

Predicted 10‐yes CVD risk, estimated using an adapted WHO/ISH‐based risk stratification was high in a large percentage of adults with hypertension in Bangladesh and was varied by age, sex, adiposity, smoking status, household wealth, and urban residence and was geographically nonuniform across districts. These results are based on predicted risk, which may not correspond with actual cardiac events, and several of these associations are partially incorporated into the risk classification. The wealth difference persisted after PSM on observed covariates, indicating that this difference cannot be attributed to compositional differences measured alone and should not be interpreted as a causal effect, as the wealth gap disappeared when adults <35 years were excluded. The hotspots suggest that predicted CVD risk is spatially clustered, although district‐level estimates are imprecise. These results suggest the consideration of risk‐focused, geographically specific, and equity‐based policies for the prevention of CVD in Bangladesh. The treatment of hypertension is not likely to be enough; interventions to address obesity, smoking, diabetes, and urban cardiometabolic environments may be necessary. Incorporating absolute risk assessment for CVD into routine primary care, with attention to hotspot districts, may help prioritize prevention efforts.

## Author Contributions

**Chowdhury Mashrur Mahdee** and **Mosiur Rhamn** designed the study, analyzed data, and wrote the first draft of this manuscript. **Anaka Tabashsum, Chowdhury Muhtadee Maheer, Fahim Shahriar, Md. Sarwar Zahan, Md. Mamunur Rashid**, and **Md. Yeasir Arafat** critically reviewed and edited the whole manuscript. All authors approved submitting the final version.

## Funding

The authors have nothing to report.

## Ethics Statement

This study used de‐identified BDHS 2022 secondary data obtained through formal requests from the DHS Program. No other ethical approval was required for the secondary analysis, as the study relied on publicly documented, anonymized survey data. The study procedures were conducted in accordance with the Declaration of Helsinki.

## Consent

The authors have nothing to report.

## Conflicts of Interest

The authors declare no conflicts of interest.

## Supporting information




**Supporting File 1**: puh270463‐sup‐0001‐SuppMat.docx.

## Data Availability

The dataset is publicly available from the DHS website: https://dhsprogram.com/data/available‐datasets.cfm.
