# Supplementary material for: Spatial and Sociodemographic Distribution of Predicted 10‐Year Cardiovascular Disease Risk Among Hypertensive Adults in Bangladesh: Evidence From BDHS 2022
Source: Public Health Chall. 2026 Nov 7;5(4):e70463. doi: 10.1002/puh2.70463 (PMC13744626; doi:10.1002/puh2.70463)
Supplement: Supplementary file 1 [file PUH2-5-e70463-s001.docx]

**Supplementary Table S1: Sensitivity analysis excluding adults aged <35 years: adapted predicted CVD risk categories across sociodemographic characteristics before and after propensity score matching, BDHS 2022**

| **Characteristics** | **Before matching** | | | | | **After matching** |
| --- | --- | --- | --- | --- | --- | --- |
|  | **n (%)** | **Adapted predicted CVD risk category, % (95% CI)** | | | | **n (%)** |
|  |  | **Low** | **Medium** | **High** | **p-value** |  |
| **Total** | 2172 | 41.07 (38.62, 43.57) | 19.69 (17.88, 21.64) | 39.23 (36.85, 41.66) | - | 1189 |
| **Age** | | | | | | |
| 35 to 54 | 1046 (48.16) | 46.83 (43.44, 50.25) | 15.15 (12.92, 17.68) | 38.02 (34.65, 41.51) | <0.001 | 510 (42.89) |
| 55 to 69 | 770 (35.45) | 34.50 (30.62, 38.59) | 23.85 (20.53, 27.52) | 41.65 (37.57, 45.85) |  | 455 (38.27) |
| 70 and above | 356 (16.39) | 38.14 (32.53, 44.10) | 24.16 (19.71, 29.26) | 37.69 (32.33, 43.37) |  | 224 (18.84) |
| **Sex** | | | | | | |
| Male | 796 (36.65) | 40.94 (37.15, 44.84) | 23.07 (19.90, 26.57) | 35.99 (32.46, 39.69) | 0.015 | 405 (34.06) |
| Female | 1376 (63.35) | 41.15 (38.03, 44.35) | 17.73 (15.59, 20.10) | 41.12 (38.07, 44.24) |  | 784 (65.94) |
| **Body Mass Index (BMI)** | | | | | | |
| Underweight | 205 (9.44) | 52.58 (44.65, 60.38) | 21.47 (15.88, 28.37) | 25.95 (19.86, 33.13) | <0.001 | 113 (9.50) |
| Normal | 664 (30.57) | 47.06 (42.66, 51.51) | 20.55 (17.23, 24.33) | 32.39 (28.41, 36.63) |  | 451 (37.93) |
| Overweight | 403 (18.55) | 44.66 (39.50, 49.93) | 15.93 (12.20, 20.53) | 39.42 (34.20, 44.89) |  | 217 (18.25) |
| Obese | 900 (41.44) | 31.94 (28.59, 35.49) | 20.29 (17.53, 23.36) | 47.77 (44.15, 51.41) |  | 408 (34.31) |
| **Smoked tobacco within 30 min before blood pressure measurement** | | | | | | |
| No | 1946 (89.59) | 43.35 (40.78, 45.96) | 17.43 (15.62, 19.40) | 39.22 (36.67, 41.84) | <0.001 | 1060 (89.15) |
| Yes | 226 (10.41) | 20.10 (15.08, 26.28) | 40.60 (33.73, 47.86) | 39.30 (32.39, 46.66) |  | 129 (10.85) |
| **Diabetes** | | | | | | |
| No | 1440 (66.30) | 60.53 (57.63, 63.35) | 29.02 (26.46, 31.72) | 10.45 (8.89, 12.26) | <0.001 | 848 (71.32) |
| Yes | 732 (33.70) | - | - | 100 |  | 341 (28.68) |
| **Hypertension** | | | | | | |
| Grade 1 | 1295 (66.24) | 68.21 (65.14, 71.13) | 7.00 (5.59, 8.74) | 24.79 (22.20, 27.57) | <0.001 | 719 (65.36) |
| Grade 2 | 465 (23.79) | - | 72.11 (67.22, 76.52) | 27.89 (23.48, 32.78) |  | 268 (24.36) |
| Grade 3 | 195 (9.97) | - | - | 100 |  | 113 (10.27) |
| **Level of education** | | | | | | |
| No education | 896 (41.25) | 43.89 (40.09, 47.76) | 22.79 (19.95, 25.91) | 33.32 (29.71, 37.14) | <0.001 | 580 (48.78) |
| Primary | 547 (25.18) | 40.89 (36.70, 45.23) | 17.84 (14.53, 21.70) | 41.27 (36.85, 45.84) |  | 338 (28.43) |
| Secondary and above | 729 (33.56) | 37.33 (33.29, 41.59) | 16.88 (13.99, 20.22) | 45.79 (41.56, 50.09) |  | 271 (22.79) |
| **Wealth index** | | | | | | |
| Poor | 887 (40.84) | 47.41 (43.76, 51.09) | 22.36 (19.62, 25.37) | 30.22 (26.96, 33.70) | <0.001 | 607 (51.05) |
| Middle | 908 (41.80) | 39.09 (35.43, 42.88) | 18.60 (15.91, 21.62) | 42.31 (38.83, 45.87) |  | 582 (48.95) |
| Rich | 377 (17.36) | 29.04 (22.82, 36.17) | 15.34 (11.56, 20.08) | 55.61 (49.06, 61.98) |  |  |
| **Division of residence** | | | | | | |
| Barishal | 215 (9.90) | 39.40 (32.39, 46.87) | 23.56 (17.77, 30.55) | 37.04 (30.25, 44.38) | 0.008 | 121 (10.18) |
| Chattogram | 297 (13.67) | 37.35 (31.78, 43.28) | 17.89 (14.11, 22.41) | 44.76 (38.94, 50.72) |  | 190 (15.98) |
| Dhaka | 276 (12.71) | 37.56 (31.62, 43.89) | 16.06 (11.99, 21.17) | 46.39 (40.14, 52.75) |  | 121 (10.18) |
| Khulna | 314 (14.46) | 40.95 (35.12, 47.05) | 22.90 (18.40, 28.13) | 36.15 (31.04, 41.59) |  | 185 (15.56) |
| Mymensingh | 206 (9.48) | 41.99 (34.50, 49.86) | 24.39 (19.38, 30.21) | 33.62 (26.11, 42.07) |  | 117 (9.84) |
| Rajshahi | 343 (15.79) | 47.90 (40.51, 55.38) | 20.03 (15.38, 25.66) | 32.07 (26.20, 38.57) |  | 187 (15.73) |
| Rangpur | 303 (13.95) | 45.72 (39.86, 51.70) | 19.96 (15.41, 25.45) | 34.32 (28.89, 40.20) |  | 162 (13.62) |
| Sylhet | 218 (10.04) | 36.22 (29.90, 43.04) | 21.06 (16.43, 26.58) | 42.72 (35.17, 50.64) |  | 106 (8.92) |
| **Region of residence** | | | | | | |
| Urban | 825 (37.98) | 34.52 (29.87, 39.49) | 16.45 (13.38, 20.07) | 49.03 (44.05, 54.02) | <0.001 | 310 (26.07) |
| Rural | 1347 (62.02) | 43.74 (40.85, 46.67) | 21.01 (18.86, 23.33) | 35.25 (32.60, 37.99) |  | 879 (73.93) |
| **Number of household members** | | | | | | |
| 1 to 4 | 1072 (49.36) | 42.36 (38.82, 45.98) | 19.39 (16.84, 22.23) | 38.25 (34.84, 41.78) | 0.166 | 568 (47.77) |
| 5 to 6 | 748 (34.44) | 40.28 (36.33, 44.36) | 21.70 (18.69, 25.04) | 38.02 (34.36, 41.83) |  | 434 (36.50) |
| 7 and above | 352 (16.21) | 38.55 (32.45, 45.02) | 16.09 (12.10, 21.08) | 45.37 (39.27, 51.60) |  | 187 (15.73) |
| **Marital status** | | | | | | |
| Never married/widowed/  divorced/separated | 522 (24.03) | 35.00 (30.49, 39.80) | 22.87 (19.02, 27.22) | 42.14 (37.31, 47.12) | 0.016 | 321 (27.00) |
| Married | 1650 (75.97) | 42.95 (40.10, 45.84) | 18.72 (16.64, 20.99) | 38.34 (35.67, 41.08) |  | 868 (73.00) |

Footnote: Adults aged <35 years were excluded from both the pre-matching sample (n = 2,172) and the matched sample (n = 1,189), which was restricted without re-matching (poor n = 607, non-poor n = 582). Percentages in the risk columns are row percentages with 95% confidence intervals; p-values are from Pearson chi-square tests (before matching only). The middle and rich wealth categories were combined as non-poor after matching. The distributions of diabetes and hypertension grade across risk categories are determined by the construction of the risk matrix (Table 1). Hypertension-grade percentages are calculated among respondents with a recorded grade (n = 1,955 before matching; n = 1,100 after matching). The tobacco item records smoking within 30 minutes before blood pressure measurement and is not current smoking status.

**Supplementary Table S2: Sensitivity analysis excluding adults aged <35 years: correlates of a higher adapted predicted CVD risk category before and after propensity score matching, BDHS 2022**

| **Characteristics** | **Before matching** | | | | **After matching** | | | |
| --- | --- | --- | --- | --- | --- | --- | --- | --- |
|  | **Adapted predicted CVD risk** | | | | **Adapted predicted CVD risk** | | | |
|  | **Unadjusted OR (95% CI)** | **p-value** | **AOR (95% CI)** | **p-value** | **Unadjusted OR (95% CI)** | **p-value** | **AOR (95% CI)** | **p-value** |
| **Age** | | | | | | | | |
| 35 to 54 | Ref. | - | Ref. | - | Ref. | - | Ref. | - |
| 55 to 69 | 1.40 (1.14, 1.71) | 0.001 | 1.63 (1.31, 2.04) | <0.001 | 1.25 (0.95, 1.63) | 0.108 | 1.43 (1.06, 1.93) | 0.021 |
| 70 and above | 1.20 (0.93, 1.54) | 0.157 | 1.65 (1.21, 2.25) | 0.002 | 1.36 (0.99, 1.88) | 0.061 | 1.76 (1.18, 2.63) | 0.006 |
| **Sex** | | | | | | | | |
| Male | Ref. | - | Ref. | - | Ref. | - | Ref. | - |
| Female | 1.10 (0.92, 1.32) | 0.279 | 1.16 (0.95, 1.43) | 0.148 | 1.26 (0.98, 1.62) | 0.068 | 1.33 (1.01, 1.75) | 0.041 |
| **Body Mass Index (BMI)** | | | | | | | | |
| Underweight | Ref. | - | Ref. | - | Ref. | - | Ref. | - |
| Normal | 1.28 (0.93, 1.77) | 0.132 | 1.25 (0.88, 1.77) | 0.218 | 1.47 (0.95, 2.30) | 0.086 | 1.54 (0.97, 2.44) | 0.069 |
| Overweight | 1.56 (1.10, 2.20) | 0.013 | 1.53 (1.06, 2.22) | 0.023 | 1.41 (0.87, 2.28) | 0.162 | 1.52 (0.92, 2.53) | 0.102 |
| Obese | 2.43 (1.77, 3.32) | <0.001 | 2.32 (1.62, 3.33) | <0.001 | 2.26 (1.47, 3.50) | <0.001 | 2.75 (1.70, 4.46) | <0.001 |
| **Smoked tobacco within 30 min before blood pressure measurement** | | | | | | | | |
| No | Ref. | - | Ref. | - | Ref. | - | Ref. | - |
| Yes | 1.54 (1.23, 1.92) | <0.001 | 1.72 (1.35, 2.20) | <0.001 | 1.74 (1.31, 2.31) | <0.001 | 1.88 (1.38, 2.57) | <0.001 |
| **Level of education** | | | | | | | | |
| No education | Ref. | - | Ref. | - | Ref. | - | Ref. | - |
| Primary | 1.25 (1.01, 1.55) | 0.041 | 1.26 (0.99, 1.59) | 0.055 | 1.15 (0.89, 1.49) | 0.294 | 1.29 (0.89, 1.85) | 0.174 |
| Secondary and above | 1.49 (1.19, 1.85) | <0.001 | 1.37 (1.06, 1.77) | 0.016 | 1.12 (0.83, 1.51) | 0.444 | 0.97 (0.64, 1.47) | 0.878 |
| **Wealth index** | | | | | | | | |
| Poor | Ref. | - | Ref. | - | Ref. | - | Ref. | - |
| Middle | 1.52 (1.25, 1.85) | <0.001 | 1.21 (0.98, 1.49) | 0.065 | 1.26 (0.99, 1.60) | 0.062 | 1.06 (0.77, 1.47) | 0.718 |
| Rich | 2.55 (1.87, 3.49) | <0.001 | 1.73 (1.23, 2.41) | 0.001 |  |  |  |  |
| **Division of residence** | | | | | | | | |
| Barishal | Ref. | - | Ref. | - | Ref. | - | Ref. | - |
| Chattogram | 1.23 (0.87, 1.74) | 0.250 | 1.14 (0.79, 1.66) | 0.480 | 1.01 (0.66, 1.54) | 0.960 | 1.07 (0.67, 1.72) | 0.782 |
| Dhaka | 1.27 (0.88, 1.82) | 0.198 | 1.10 (0.75, 1.62) | 0.617 | 0.93 (0.56, 1.53) | 0.764 | 1.09 (0.63, 1.89) | 0.753 |
| Khulna | 0.95 (0.68, 1.33) | 0.770 | 0.88 (0.62, 1.26) | 0.494 | 0.79 (0.50, 1.24) | 0.300 | 0.86 (0.51, 1.46) | 0.588 |
| Mymensingh | 0.89 (0.60, 1.32) | 0.551 | 0.97 (0.64, 1.47) | 0.900 | 0.92 (0.56, 1.51) | 0.746 | 0.96 (0.56, 1.65) | 0.875 |
| Rajshahi | 0.75 (0.52, 1.10) | 0.141 | 0.80 (0.54, 1.19) | 0.269 | 0.67 (0.40, 1.10) | 0.112 | 0.73 (0.42, 1.26) | 0.255 |
| Rangpur | 0.83 (0.59, 1.16) | 0.275 | 0.94 (0.65, 1.35) | 0.822 | 0.89 (0.54, 1.45) | 0.626 | 0.98 (0.76, 1.29) | 0.946 |
| Sylhet | 1.20 (0.82, 1.76) | 0.351 | 1.14 (0.77, 1.71) | 0.509 | 0.89 (0.55, 1.45) | 0.638 | 1.01 (0.58, 1.74) | 0.974 |
| **Region of residence** | | | | | | | | |
| Urban | Ref. | - | Ref. | - | Ref. | - | Ref. | - |
| Rural | 0.61 (0.49, 0.77) | <0.001 | 0.79 (0.63, 0.99) | 0.043 | 0.75 (0.57, 0.99) | 0.041 | 0.71 (0.53, 0.95) | 0.023 |
| **Number of household members** | | | | | | | | |
| 1 to 4 | Ref. | - | Ref. | - | Ref. | - | Ref. | - |
| 5 to 6 | 1.04 (0.86, 1.27) | 0.692 | 0.97 (0.79, 1.18) | 0.758 | 1.11 (0.85, 1.43) | 0.443 | 0.99 (0.76, 1.29) | 0.943 |
| 7 and above | 1.26 (0.95, 1.68) | 0.114 | 1.00 (0.75, 1.34) | 0.980 | 0.98 (0.69, 1.40) | 0.916 | 0.74 (0.51, 1.08) | 0.116 |
| **Marital status** | | | | | | | | |
| Never married/widowed/  divorced/separated | Ref. | - | Ref. | - | Ref. | - | Ref. | - |
| Married | 0.79 (0.64, 0.96) | 0.019 | 0.82 (0.64, 1.06) | 0.124 | 0.73 (0.57, 0.94) | 0.016 | 0.80 (0.59, 1.08) | 0.148 |
| **Interaction effect** | | | | | | | | |
| Non-poor (vs. Poor) within Primary | - | - | - | - | - | - | 0.95 (0.57, 1.58) | 0.842 |
| Non-poor (vs. Poor) within Secondary and above | - | - | - | - | - | - | 2.38 (1.30, 4.36) | 0.005 |

Footnote: OR, odds ratio; AOR, adjusted odds ratio; CI, confidence interval; Ref., reference. Adults aged <35 years were excluded, so the age reference group is 35 to 54 years. Middle and rich wealth categories were combined as non-poor after matching (matched columns: “Middle” = non-poor vs. poor). The tobacco item records smoking within 30 minutes before blood pressure measurement, not current smoking status. Age, sex, BMI and this item contributed to outcome construction, so their coefficients are descriptive correlates, not independent predictors of incident CVD. PSM and interaction estimates are non-causal and susceptible to residual confounding.

**Supplementary Table S3: Standardized mean differences (SMDs) before and after 1:1 nearest-neighbor propensity score matching (non-poor vs. poor), BDHS 2022**

| **Characteristics** | **Standardized mean differences** | |
| --- | --- | --- |
|  | **Before Matching** | **After Matching** |
| **Age** | | |
| <35 | 0.0308 | 0.0990 |
| 35 to 54 | 0.0985 | -0.0858 |
| 55 to 69 | -0.0212 | 0.0093 |
| 70 and above | -0.1544 | 0.0087 |
| **Sex** | | |
| Male | 0.1108 | 0.0407 |
| Female | -0.1108 | -0.0407 |
| **Division** | | |
| Barishal | -0.1664 | 0.0052 |
| Chattogram | 0.0446 | -0.0080 |
| Dhaka | 0.2606 | 0.0230 |
| Khulna | 0.0990 | -0.0156 |
| Mymensingh | -0.2313 | 0.0165 |
| Rajshahi | -0.0568 | -0.0640 |
| Rangpur | -0.1802 | 0.0178 |
| Sylhet | 0.1044 | 0.0348 |
| **Residence** | | |
| Urban | 0.7125 | 0.0852 |
| Rural | -0.7125 | -0.0852 |
| **Marital** | | |
| Never/widowed/divorced/separated | -0.0451 | 0.0067 |
| Married | 0.0451 | -0.0067 |
| **BMI** | | |
| Underweight | -0.4128 | 0.0129 |
| Normal | -0.3885 | -0.0236 |
| Overweight | 0.0610 | 0.0532 |
| Obese | 0.4595 | -0.0284 |
| **Education** | | |
| None | -0.5900 | -0.0649 |
| Primary | -0.0764 | 0.0434 |
| Secondary and above | 0.5801 | 0.0198 |

Footnote: SMDs compare non-poor with poor respondents; an absolute SMD <0.10 indicates acceptable balance. Both categories of the binary covariates (sex, residence, marital status) are listed; Figure 1 displays one category of each.
